# Association of change in total cholesterol level with mortality: A population-based study

**DOI:** 10.1371/journal.pone.0196030

**Published:** 2018-04-19

**Authors:** Su-Min Jeong, Seulggie Choi, Kyuwoong Kim, Sung-Min Kim, Gyeongsil Lee, Joung Sik Son, Jae-Moon Yun, Sang Min Park

**Affiliations:** 1 Department of Family Medicine, Seoul National University Hospital, Seoul, Republic of Korea; 2 Department of Biomedical Sciences, Seoul National University Graduate School, Seoul, Republic of Korea; 3 Department of Family Medicine, Seoul National University College of Medicine, Seoul, Korea, Seoul, Republic of Korea; University of Tampere, FINLAND

## Abstract

**Background:**

Hypercholesterolemia is a well-established risk factor for coronary heart disease, but the association between cholesterol level change and mortality is not fully understood. We aimed to investigate the association of 2 year (2002–2003 to 2004–2005) change in cholesterol with all-cause and cause-specific mortality in a population-based cohort study.

**Methods and findings:**

The study population consisted of 269,391 participants aged more than 40 years who were free of myocardial infarction, stroke and cancer using the Korean National Health Insurance Service—National Health Screening Cohort. Cholesterol levels were classified into 1st, 2nd and 3rd tertiles during each of the first and second health examinations, respectively. The participants were followed-up for all-cause and cause-specific mortality from 1 January 2006 to 31 December 2013. Compared to participants who stayed within the 2nd tertile group for cholesterol during both the first and second examinations, participants who became or maintained cholesterol levels to the 1st tertile during the second examination had increased risk of all-cause mortality [adjusted hazard ratio (aHR) with 95% confidence interval (95% CI) = 1.28 (1.18–1.38) in 1st/1st, 1.16 (1.07–1.26) in 2nd/1st and 1.47 (1.32–1.64) in 3rd/1st tertile levels, respectively]. In addition, increased or persistent high cholesterol levels to the 3rd tertile was associated with elevated risk for all-cause mortality [aHR (95% CI) = 1.10 (1.01–1.20) in 1st/2nd, 1.16(1.03–1.31) in 1st/3rd and 1.15(1.05–1.25) in 3rd/3rd tertile levels].

**Conclusions:**

Changes in cholesterol levels in either direction to low cholesterol or persistently low cholesterol levels were associated with higher risk of mortality. Particularly, spontaneous decline in cholesterol levels may be a marker for worsening health conditions.

## Introduction

Hypercholesterolemia is a well-established risk factor for coronary heart disease (CHD) [1, 2], indicating that cholesterol lowering therapy would be beneficial for prevention of CHD [3]. However, previous studies on the association between cholesterol levels and mortality have shown inconsistent results that vary according to age, cause of death, and sex [4–8]. Previous findings indicate that that low cholesterol was associated with high mortality [6, 7]. For example, among the elderly, frailty or poor health status may have contributed to the increased risk of death among those with low cholesterol levels [9]. In addition, other previous studies have revealed a U-shaped relationship between cholesterol and mortality [10, 11]. Interestingly, the increased risk of death among those with low cholesterol was due to non-vascular disease, such as liver disease [12] or cancer [13], which is in contrast to the high contribution of cardiovascular disease (CVD) related mortality among those with high cholesterol levels [11].

A number of studies investigated the association between the change in cholesterol levels and mortality [5, 8]. A study from the Honolulu Heart Program revealed a 30% increase in mortality among people who had decreased levels of cholesterol [5]. The most common causes of deaths among those with declines in cholesterol levels were malignancies of the hematopoietic system, esophagus, and prostate and non-malignant liver disease. However, this study was limited to a relatively small number of male subjects (n = 5,941). In a study using the Framingham data, those with decreasing cholesterol levels were associated with increased risk of all-cause and CVD mortality [8]. Spontaneous decline in cholesterol of 14 mg/dL during 14-years was associated with 11% increased risk of mortality, compared to those with stable or increased cholesterol levels. However, previous studies have been mainly performed in the Western population and the number of study population is relatively small.

In this study, we aimed to elucidate the association between change in cholesterol and all-cause mortality as well as cause-specific mortality using a nationally representative cohort.

## Methods

### Data source

National Health Insurance Service (NHIS) in the Republic of Korea covers approximately 97% of the Korean population and provides biennial health screening examinations called the National Health Screening Program (NHSP) to all enrollees above 40 years old [14]. The NHSP offers screening tests for several conditions, including anemia, liver disease and kidney disease as well as cardiovascular risk factors including blood pressure, lipid profile and fasting glucose. The National Health Insurance Service—National Health Screening Cohort (NHIS-HEALS) database is composed of demographic factors, results from the NHSP, utilization of medical facilities at outpatient and inpatient settings with the International Classification of Diseases, 10th revision (ICD-10) codes and the date and cause of death from the Korea National Statistical Office. In addition, NHIS-HEALS contains demographic information such as age, gender, health insurance premium, and disability. The NHIS database has been widely used in multiple epidemiological studies [15, 16] and its validity is described in detail elsewhere [17].

### Study population

**Fig 1:**
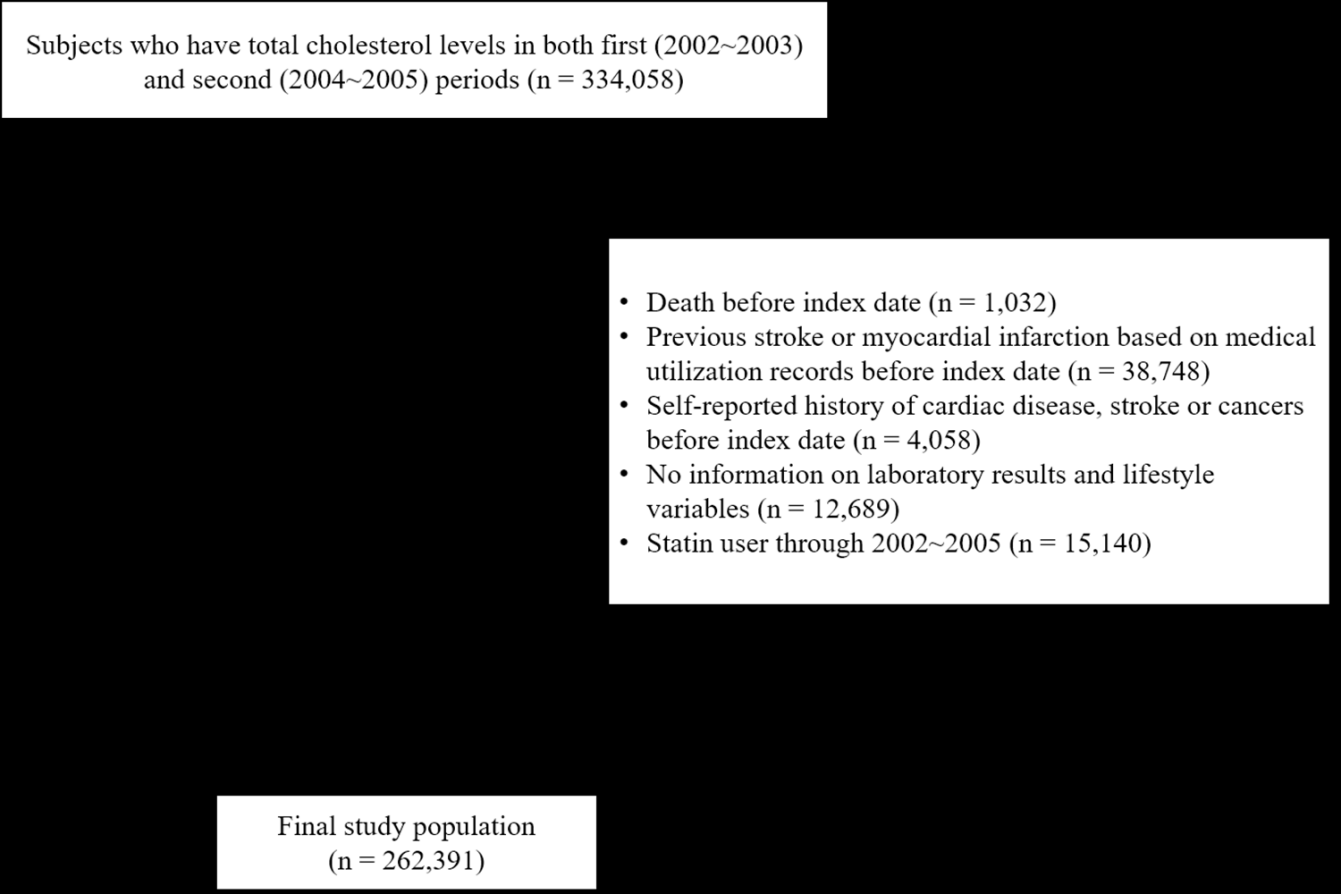
Flow chart of the study population selection.

The NHIS-HEALS database consists of about 510,000 randomly selected participants among the general population over 40 years ages who underwent health screening examinations in 2002 or 2003 and followed-up until 31 December 2013. We identified 334,058 participants who had cholesterol values in the first (2002–2003) and second (2004–2005) health examination periods. We excluded those who passed away (n = 1,032) before the index date of 1 January 2006. In addition, we also excluded those who were diagnosed with myocardial infarction (MI) or stroke (n = 38,748), reported a history of MI, stroke or cancer before the index date (n = 4,058) or had missing data on laboratory results and lifestyle variables (n = 12,689). Furthermore, we excluded statin users (n = 15,140), who were defined as those with more than 30 cumulative defined daily dose (cDDD) of statins during 2002 to 2005 to investigate the association between spontaneous change of cholesterol levels and mortality, since we assumed that decreased cholesterol with statins or without statins may be differently associated with mortality [18]. Therefore, final study population consisted of 262,391 participants (Fig 1).

This study was approved by Seoul National University Hospital’s institutional review board (IRB number: 1703-039-863), and consent from individual patients was waived as NHIS-HEALS was anonymized according to strict confidentiality guidelines. This study was approved by NHIS (NHIS-2017-2-456)

### Change in cholesterol level

Total cholesterol levels were measured at each health examination after fasting for at least 8 hours. Baseline (2002–2003) cholesterol levels were classified into 1st (< 182 mg/dL), 2nd (182–212 mg/dL) and 3rd tertiles (≥ 212 mg/dL). In addition, follow-up (2004–2005) cholesterol levels were classified into 1st tertile (< 181 mg/dL), 2nd tertile (181–210 mg/dL) and 3rd tertile (≥ 211 mg/dL). Participants who stayed in the 2nd tertile group for both the initial and second health examination periods were considered the reference group.

### Outcome assessment

Deaths from 1 January 2006 to 31 December 2013 were determined by using death certificates from the National Statistical Office of Korea. The causes of deaths were categorized into CVD (ICD-10 codes I00-I99) and cancer (ICD-10 C00-C97) according to the ICD-10 codes.

### Covariates

Body mass index (BMI) was calculated by weight (kg) divided by height (m) squared and grouped into < 25 kg/m^2^ and ≥ 25 kg/m^2^ according to Asian-Pacific cut-off points [19]. Smoking status was classified into never, former and current smokers. Alcohol consumption was divided into none or at least one drink per week. Insurance premium divided into quintiles was used to classify household income status. The presence of disability was based on National Registration for Disability. Comorbidities were summarized by the Charlson comorbidity index (CCI) [20] using ICD-10 codes from 2002 to 2005. History of hypertension and diabetes were defined as having reported being previously diagnosed with or taking anti-hypertensive or anti-diabetic medications.

### Statistical analyses

Analysis of variance (ANOVA) for continuous variables and Chi-squared test for categorical variables were used to compare baseline characteristics according to baseline cholesterol levels. Continuous variables are expressed as mean [standard deviation (SD)]. We used the Cox-regression hazard model to evaluate the risk of mortality according to the change in cholesterol levels. We adjusted for age and sex in model 1. In model 2, we additionally adjusted life style variables (BMI, smoking status and drinking status), socioeconomic factors (household income and disability) and medical conditions (CCI, history of hypertension and diabetes, systolic blood pressure and fasting glucose level). We performed sensitivity analysis by excluding deaths that occurred within the first two years from the index date to account for the possibility of reverse-causality. All data processing and statistical analyses were carried out using SAS 9.4 (SAS Institute, Cary, NC) and STATA 14.1 (Stata Corp, College Station, TX, USA), respectively.

## Results

### Baseline characteristics

The mean age of the total population was 54 (SD 8.8) years and were composed of 153,759 (58.6%) male subjects (Table 1). The mean cholesterol level at baseline was 198.0 (SD 36.1) mg/dL in 2002–2003 and 196.6 (SD 35.2) mg/dL in 2004–2005 with 0.57 (Standard error, 0.001) correlation coefficient for cholesterol. Subjects in the baseline 3rd tertile cholesterol levels were more likely to be old, female, obese (≥ 25 kg/m^2^) and to have higher systolic blood pressure and fasting blood glucose compared to those in the 1st and 2nd tertiles for cholesterol.

**Table 1:** Baseline characteristics of study population.

a	Total	1^st^ tertile (< 182 mg/dL)	2^nd^ tertile (182–212 mg/dL)	3^rd^ tertile (≥ 212 mg/dL)	*p*-value
All subjects ^a^, N (%)	262,391 (100)	88,486 (33.7)	87,244 (33.3)	86,661 (33.0)	
Age, mean (SD), years	54.0 (8.8)	53.3 (9.0)	53.8 (8.7)	54.7 (8.7)	< 0.001
< 65	223,807 (85.3)	75,802 (85.7)	74,885 (85.8)	73,120 (84.4)	
≥ 65	13,541 (14.7)	12,684 (14.3)	12,359 (14.2)	13,541 (15.6)	
Sex, N (%)					< 0.001
Male	153,759 (58.6)	52,296 (59.1)	51,669 (59.2)	49,794 (57.5)	
Female	108,632 (41.4)	36,190 (40.9)	35,575 (40.8)	36,867 (42.5)	
Baseline TC, mean (SD), mg/dL	198.0 (36.1)	160.6 (16.3)	196.2 (8.5)	237.9 (24.3)	
Body mass index, N (%), kg/m^2^					< 0.001
< 25	178,144 (67.9)	65,451 (74.0)	58,986 (67.6)	53,707 (62.0)	
≥ 25	84,247 (32.1)	23,035 (26.0)	28,258 (32.4)	32,954 (38.0)	
Smoking status, N (%)					< 0.001
Never	176,035 (67.1)	59,705 (67.5)	58,455 (67.0)	57,875 (66.8)	
Former	25,593 (9.7)	8,293 (9.4)	8,631 (9.9)	8,669 (10.0)	
Current	60,763 (23.2)	20,488 (23.1)	20,158 (23.1)	20,117 (23.2)	
Drinking, N (%)					< 0.001
No	144,737 (55.2)	48,850 (55.2)	47,691 (54.7)	48,196 (55.6)	
Yes	117,654 (44.8)	39,636 (44.8)	39,553 (45.3)	38,465 (44.4)	
Household income status, N (%)					< 0.001
1st Quintile (low) & medical aids	37,413 (14.3)	12,645 (14.3)	12,022 (13.8)	12,746 (14.7)	
2nd Quintile	36,832 (14.0)	13,001 (14.7)	12,113 (13.9)	11,718 (13.5)	
3rd Quintile	39,167 (14.9)	13,748 (15.5)	12,847 (14.7)	12,572 (14.5)	
4th Quintile	53,299 (20.3)	18,191 (20.6)	17,671 (20.2)	17,437 (20.1)	
5th Quintile (high)	95,680 (36.5)	30,901 (34.9)	32,591 (37.4)	32,188 (37.1)	
Disability, N (%)					< 0.001
No	261,082 (99.5)	87,978 (99.4)	86,858 (99.6)	86,246 (99.5)	
Yes	1,309 (0.5)	508 (0.6)	386 (0.4)	415 (0.5)	
Charlson comorbidity index, N (%)					< 0.001
< 4	246,101 (93.8)	82,596 (93.3)	82,067 (94.1)	81,437 (94.0)	
≥ 4	16,290 (6.2)	5,889 (6.7)	5,177 (5.9)	5,224 (6.0)	
Hypertension, N (%)					< 0.001
No	241,454 (92.0)	82,412 (93.1)	80,263 (92.0)	78,779 (90.9)	
Yes	20,937 (8.0)	6,074 (6.9)	6,981 (8.0)	7,882 (9.1)	
Diabetes, N (%)					0.004
No	253,176 (96.5)	85,414 (96.5)	84,287 (96.6)	83,475 (96.3)	
Yes	9,215 (3.5)	3,072 (3.5)	2,957 (3.4)	3,186 (3.7)	
Systolic blood pressure, mean(SD), mmHg	125.8 (16.9)	124.2 (16.9)	125.7 (16.8)	127.4 (17.0)	< 0.001
Fasting blood glucose, mean(SD), mg/dL	96.8 (27.5)	95.5 (26.9)	96.4 (26.9)	98.5 (28.7)	< 0.001

TC, Total cholesterol;

^a^ The subjects were divided into three groups according to tertiles of cholesterol levels at baseline (2002–2003).

### Change in cholesterol and mortality

The mean follow-up period was 8.0 years and the mean time to death was 4.7 years. There was a U-shaped relationship between baseline cholesterol levels and all-cause mortality [adjusted HR (aHR) = 1.13; 95% confidence interval (CI) = 1.06–1.20 in 1st tertile, aHR = 1.15; 95% CI = 1.08–1.22 in 3rd tertile], compared to 2nd tertile at baseline (S1 Table) (S1 Fig).

Subjects with persistent 1st tertile cholesterol levels and increasing cholesterol levels from the 1st tertile to the 2nd and 3rd tertile levels during the follow-up period were associated with increased risk of all-cause mortality [aHR (95% CI) = 1.28 (1.18–1.38), 1.10 (1.01–1.20) and 1.16 (1.03–1.31), respectively] compared to those with persistent 2nd tertile levels (Table 2). Subjects with decreasing cholesterol levels from 3rd tertile levels to 1st and 2nd and persistent 3rd tertile levels were associated with increased risk of all-cause mortality [aHR (95% CI) = 1.47 (1.32–1.64), 1.15 (1.05–1.26) and 1.15 (1.05–1.25), respectively] compared to those with persistent 2nd tertile levels. Decreasing cholesterol from 2nd tertile to 1st tertile levels was associated with increased risk of all-cause mortality [aHR (95% CI) = 1.16 (1.07–1.26)]. These associations were prominent in groups less than 65 years old or men (S2 Table). Among statin users (n = 15,140), those with persistent 1st tertile cholesterol levels were associated with high all-cause mortality. (S3 Table).

**Table 2:** Hazard ratios for mortality by change of total cholesterol.

Baseline TC	1st tertile			2nd tertile			3rd tertile		
	(< 182 mg/dL)			(182–212 mg/dL)			(≥ 212 mg/dL)		
Follow-up TC (tertile) ^a^	1st	2nd	3rd	1st	2nd	3rd	1st	2nd	3rd
Total number (%)	55,248 (62.4)	25,050 (28.3)	8,188 (9.3)	24,853 (28.5)	38,289 (43.9)	24,102 (27.6)	8,186 (9.4)	25,359 (29.3)	53,116 (61.3)
All-cause mortality									
Cases (N)	2,954	1,062	386	1,114	1,316	861	480	1,000	1,942
Unadjusted HR	1.57	1.24	1.38	1.31	1.00	1.04	1.73	1.15	1.07
(95% CI)	(1.47–1.68)	(1.14–1.34)	(1.24–1.55)	(1.21–1.42)		(0.96–1.13)	(1.55–1.92)	(1.06–1.25)	(0.99–1.14)
*p*-value	< 0.001	< 0.001	< 0.001	< 0.001		0.36	< 0.001	0.001	0.072
Model 1^b^									
aHR	1.46	1.22	1.31	1.19	1.00	1.05	1.43	1.07	1.05
(95% CI)	(1.37–1.55)	(1.12–1.32)	(1.17–1.47)	(1.10–1.29)		(0.96–1.14)	(1.29–1.59)	(0.99–1.17)	(0.98–1.13)
*p*-value	< 0.001	< 0.001	< 0.001	< 0.001		0.288	< 0.001	0.085	0.153
Model 2^c^									
aHR	1.28	1.1	1.16	1.16	1.00	1.03	1.47	1.15	1.15
(95% CI)	(1.18–1.38)	(1.01–1.20)	(1.03–1.31)	(1.07–1.26)		(0.95–1.13)	(1.32–1.64)	(1.05–1.26)	(1.05–1.25)
*p*-value	< 0.001	0.034	0.013	< 0.001		0.462	< 0.001	0.003	0.002
CVD mortality									
Cases (N)	379	149	64	154	188	152	78	177	403
Unadjusted HR	1.41	1.22	1.61	1.27	1.00	1.28	1.97	1.43	1.55
(95% CI)	(1.19–1.68)	(0.98–1.51)	(1.21–2.13)	(1.03–1.57)		(1.04–1.59)	(1.51–2.56)	(1.16–1.75)	(1.30–1.84)
*p*-value	< 0.001	0.073	0.001	0.028		0.021	< 0.001	0.001	< 0.001
Model 1^b^									
aHR	1.3	1.18	1.47	1.14	1.00	1.27	1.57	1.3	1.48
(95% CI)	(1.09–1.55)	(0.95–1.47)	(1.11–1.96)	(0.92–1.42)		(1.02–1.57)	(1.21–2.05)	(1.06–1.60)	(1.24–1.76)
*p*-value	0.003	0.127	0.008	0.217		0.031	0.001	0.012	< 0.001
Model 2^c^									
aHR	1.37	1.22	1.47	1.13	1.00	1.22	1.43	1.2	1.3
(95% CI)	(1.12–1.67)	(0.97–1.53)	(1.10–1.98)	(0.91–1.39)		(0.98–1.51)	(1.08–1.89)	(0.96–1.50)	(1.05–1.61)
*p*-value	0.002	0.094	0.01	0.259		0.073	0.012	0.114	0.014
Cancer mortality									
Cases (N)	1,392	501	140	487	613	357	189	445	814
Unadjusted HR	1.59	1.26	1.08	1.23	1.00	0.93	1.46	1.1	0.96
(95% CI)	(1.45–1.75)	(1.12–1.41)	(0.90–1.30)	(1.09–1.39)		(0.81–1.06)	(1.24–1.72)	(0.97–1.24)	(0.86–1.07)
*p*-value	< 0.001	< 0.001	0.418	0.001		0.258	< 0.001	0.12	0.449
Model 1^b^									
aHR	1.48	1.24	1.04	1.13	1.00	0.94	1.23	1.04	0.96
(95% CI)	(1.35–1.63)	(1.11–1.40)	(0.87–1.25)	(1.00–1.27)		(0.83–1.07)	(1.05–1.45)	(0.92–1.17)	(0.86–1.07)
*p*-value	< 0.001	< 0.001	0.648	0.044		0.371	0.012	0.549	0.447
Model 2^c^									
aHR	1.34	1.15	0.94	1.1	1.00	0.93	1.26	1.1	1.03
(95% CI)	(1.19–1.50)	(1.01–1.31)	(0.78–1.14)	(0.98–1.24)		(0.82–1.06)	(1.06–1.50)	(0.96–1.25)	(0.90–1.17)
*p*-value	< 0.001	0.037	0.546	0.108		0.269	0.009	0.192	0.672

TC, total cholesterol; CVD, cardiovascular disease; HR, hazard ratio; CI, confidence interval.

^a^ Cholesterol levels at follow-up were divided into three groups according to tertiles; 1^st^ tertile (< 181 mg/dL), 2^nd^ tertile (181–210 mg/dL) and 3^rd^ tertile (≥ 211 mg/dL).

^b^ Adjusted for age and sex in model 1.

^c^ Adjusted for age, sex, body mass index, baseline total cholesterol, systolic blood pressure, fasting blood glucose, hypertension, diabetes, Charlson comorbidity index, alcohol drinking, smoking status, disability and household income in model 2.

The risk of CVD mortality was elevated in those with persistent 3rd tertile levels and increasing cholesterol levels from 1st tertile to 3rd tertile levels [aHR (95% CI) = 1.30 (1.05–1.61) and 1.47 (1.10–1.98), respectively]. Subjects with persistent 1st tertile levels and decreasing cholesterol levels from 3rd tertile to 1st tertile levels were associated with high CVD mortality risk [aHR (95% CI) 1.37 (1.12–1.67) and 1.43 (1.08–1.89), respectively].

Increased risk of cancer mortality was observed those in persistent 1st tertile levels and decreasing cholesterol levels from 3rd tertile to 1st tertile levels [aHR (95% CI) = 1.34 (1.19–1.50) and 1.26 (1.06–1.50)].

### Sensitivity analysis

In the sensitivity analysis of excluding deaths within the first two years of follow-up (n = 2,069), increased risks of all-cause mortality, CVD mortality and cancer mortality were consistently observed in subjects with persistent 1st tertile levels [aHR (95% CI) = 1.27 (1.17–1.39), 1.36 (1.10–1.69) and 1.35 (1.19–1.53)] compared to those with persistent 2nd tertile levels (Table 3). Moreover, decreasing cholesterol levels from 2nd and 3rd tertile to 1st tertile levels were significantly associated with high all-cause mortality risk. On the other hand, increasing cholesterol levels from 1st tertile to 2nd and 3rd tertile levels were associated with high all-cause mortality [aHR (95% CI) = 1.11 (1.01–1.22) and 1.15 (1.01–1.31)].

**Table 3:** Sensitivity analysis for mortality after excluding deaths within 2 years from index date.

Baseline TC	1st tertile			2nd tertile			3rd tertile		
	(< 182 mg/dL)			(182–212 mg/dL)			(≥ 212 mg/dL)		
Follow-up TC ^a^	1st	2nd	3rd	1st	2st	3rd	1st	2nd	3rd
All-cause mortality									
Cases (N)	2,361	873	309	893	1,107	712	387	812	1,592
Adjusted HR ^b^	1.27	1.11	1.15	1.11	1.00	1.01	1.37	1.07	1.07
(95% CI)	(1.17–1.39)	(1.01–1.22)	(1.01–1.31)	(1.01–1.21)		(0.92–1.11)	(1.21–1.55)	(0.97–1.18)	(0.97–1.17)
*p*-value	< 0.001	0.033	0.036	0.022		0.813	< 0.001	0.187	0.169
CVD mortality									
Cases (N)	2,045	755	258	768	944	585	322	668	1,259
Adjusted HR ^b^	1.36	1.14	1.39	1.06	1.00	1.17	1.32	1.08	1.18
(95% CI)	(1.10–1.69)	(0.89–1.47)	(1.00–1.93)	(0.84–1.33)		(0.92–1.47)	(0.97–1.79)	(0.85–1.38)	(0.94–1.49)
*p*-value	0.005	0.306	0.047	0.651		0.193	0.074	0.525	0.147
Cancer mortality									
Cases (N)	297	146	11	169	501	105	25	112	124
Adjusted HR ^b^	1.35	1.17	0.92	1.07	1.00	0.93	1.2	1.02	0.96
(95% CI)	(1.19–1.53)	(1.02–1.35)	(0.74–1.14)	(0.94–1.22)		(0.81–1.08)	(0.99–1.45)	(0.88–1.19)	(0.84–1.11)
*p*-value	< 0.001	0.026	0.421	0.313		0.334	0.064	0.778	0.611

TC, total cholesterol; CVD, cardiovascular disease; HR, hazard ratio; CI, confidence interval.

^a^ Cholesterol levels at follow-up were divided into three groups according to tertiles; 1^st^ tertile (< 181 mg/dL), 2^nd^ tertile (181–210 mg/dL) and 3^rd^ tertile (≥ 211 mg/dL).

^b^ Adjusted for age, sex, body mass index, baseline total cholesterol, statin medication, systolic blood pressure, fasting blood glucose, hypertension, diabetes, Charlson comorbidity index, alcohol drinking, smoking status, disability and household income.

## Discussion

We demonstrated that subjects with both decreasing cholesterol levels and persistently low cholesterol levels were significantly associated with an increased risk of all-cause mortality compared to those remaining at a stable middle cholesterol level. In addition, subjects with increasing cholesterol levels and persistently high cholesterol were associated with high all-cause mortality risk. CVD mortality according to change in cholesterol showed similar patterns with all-cause mortality. On the other hand, cancer mortality risk was elevated in those with persistently low and decreasing cholesterol levels.

Recent studies have found an inverse association between cholesterol level and mortality [9, 21–24]. However, it is uncertain whether low cholesterol level contributes to increased risk of mortality or is rather a surrogate marker for other serious illnesses. A longitudinal study in Denmark showed that severe diseases that could lower total cholesterol might increase mortality [21]. In addition, high cholesterol was associated with lower non-CVD mortality such as cancer mortality in subjects with age ≥ 65 years [23]. A study in patients with end stage renal disease suggested that systemic inflammation and malnutrition may explain this inverse association through cytokines related to acute or chronic inflammation which contribute low cholesterol levels and higher mortality [25].

Decline in cholesterol levels from high to low tertile within 2 years might be related to conditions that can alter cholesterol homeostasis including uptake, synthesis and storage. Furthermore, decline in cholesterol levels consequently can lead increased susceptibility to fatal diseases [9] by dysfunction in cellular functions [26]. In our study, cancer mortality was higher in those with sustained low tertile of cholesterol levels or steep decline (from 3rd to1st tertile levels) within 2 years. Increased lipid uptake in cancer cells may account for this steep decline. Cancer cells have an increasing requirement for lipids in order to reduce the fluidity of cell membranes and increase chemotherapy resistance by saturating the cell membrane with lipid, thereby increasing their chances of survival [27].

Besides cancer mortality, low total cholesterol was associated with high CVD mortality in prospective cohort studies [10, 28, 29]. This study also showed that low cholesterol levels as well as high cholesterol levels were associated with high CVD mortality. We could not verify the exact reasons for this result, but many previous studies have suggested that 1) this is due to chance findings or reverse epidemiology [10]; 2) the effects of malnutrition, a risk factor for non-ischemic heart disease [28]; 3) subfractions, particularly for low levels of high density lipoprotein-cholesterol (HDL-C) [29]. In patients with acute MI, significant decreases in total cholesterol, low density lipoprotein-cholesterol (LDL-C), and HDL-C levels were reported, although the causes were not clear [30].

There are several limitations in our study that need to be considered. First, we could not evaluate lipoprotein subfractions such as HDL-C and LDL-C, due the lack of data [22]. Second, possibility of day-to day variability due to laboratory error or biologic variability may exist, as we used single cholesterol measurement. Repeated measurement would be more accurate to classify the subjects [31]. Furthermore, while there might be possibility of increasing or decreasing trends of cholesterol levels due to life style change in Korean population, such as change of dietary patterns or increasing trends of obesity, we used classification of tertiles for first and second examination, separately and there was no substantial change in average cholesterol from 1998 to 2010 in Korean [32]. Third, we cannot rule out the possibility of reverse causality. Although we have excluded deaths within first 2 years from index date to resolve reverse causality, there might be other predisposing conditions that can cause steep decline of cholesterol in short periods. Fourth, the changes in cholesterol levels may reflect a ‘regression to the mean’ rather than any biologic effect [33].

In conclusion, decreasing cholesterol levels or persistently low cholesterol levels were associated with higher risk of all-cause, cancer and CVD mortality. In addition, increasing cholesterol levels or persistently high cholesterol levels was also associated with high CVD mortality risk. This suggests that decreased cholesterol and low cholesterol levels may be an indicator for poor health status. The clinical implication of this study is that individuals with spontaneously decreased cholesterol or persistently low cholesterol levels are at increased risk of mortality and may require careful attention for signs of deterioration of health [8].

## Supporting information

S1 Table Hazard ratios for mortality by tertiles of baseline total cholesterol. (DOCX)

S2 Table Stratified, multivariate-adjusted analysis of all-cause mortality risk by change in total cholesterol. (DOCX)

S3 Table The association between mortality and cholesterol change among subjects with statin medication. (DOCX)

S1 Fig The association between baseline cholesterol levels and mortality. (a) All-cause mortality (b) Cardiovascular disease mortality and (C) Cancer mortality according to the tertiles of baseline cholesterol showed U-shaped association.
(TIF)
