# Correction: Association of change in total cholesterol level with mortality: A population-based study

**DOI:** 10.1371/journal.pone.0215934

**Published:** 2019-04-18

**Authors:** Su-Min Jeong, Seulggie Choi, Kyuwoong Kim, Sung-Min Kim, Gyeongsil Lee, Joung Sik Son, Jae-Moon Yun, Sang Min Park

Fig 1, “Flow chart of the study population selection,” is incorrect. Please see the correct Fig 1 here.

**Fig 1:**
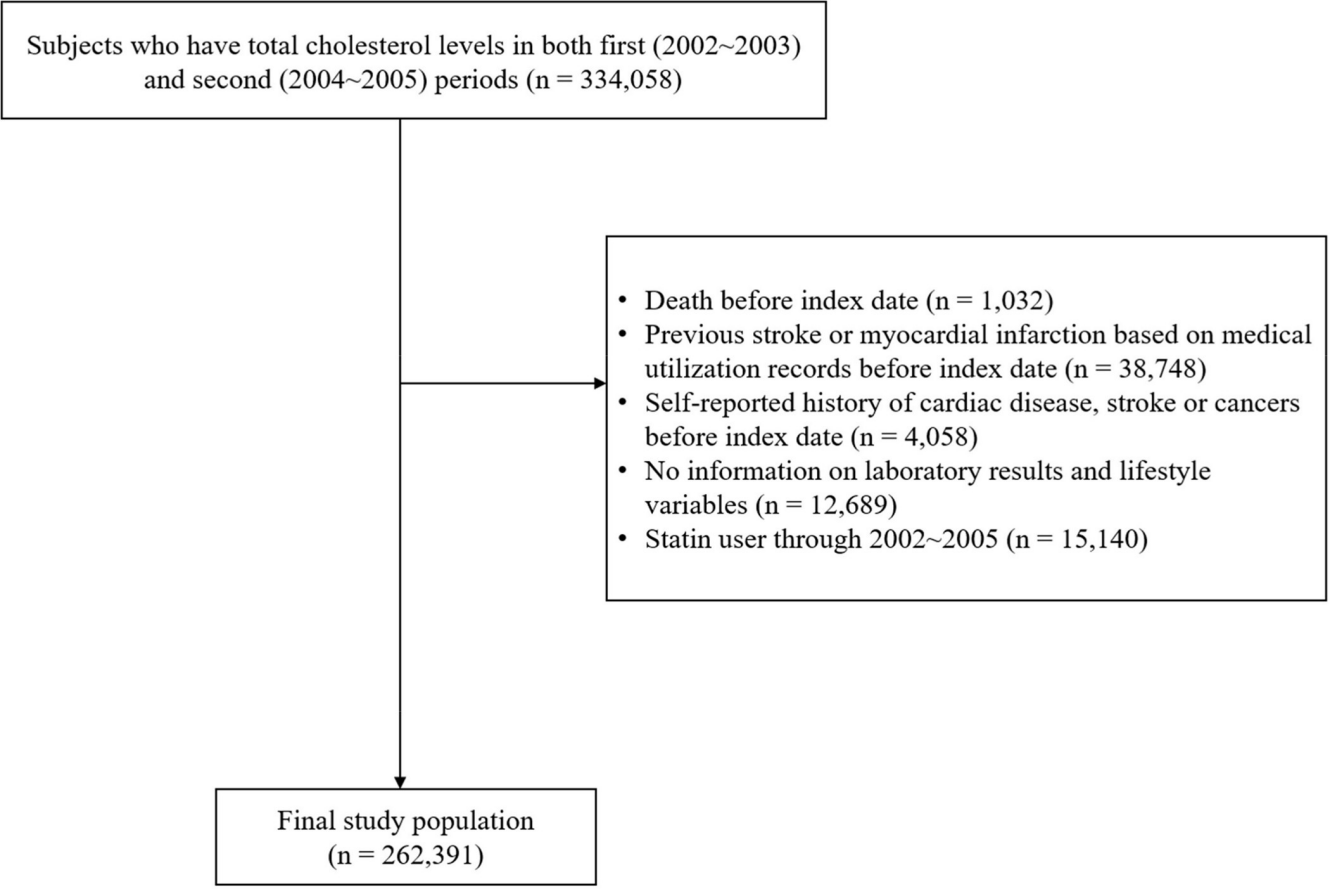
Flow chart of the study population selection.
