# Supplementary material for: Association of change in total cholesterol level with mortality: A population-based study
Source: PLoS One. 2018 Apr 19;13(4):e0196030. doi: 10.1371/journal.pone.0196030 (PMC5908176; doi:10.1371/journal.pone.0196030)
Supplement: S1 Table — (DOCX) [file pone.0196030.s001.docx]

**S1 Table.** Hazard Ratios for mortality by tertiles of baseline total cholesterol

| Baseline TC | 1^st^ tertile  (< 182 mg/dL) | *p*- value | 2nd tertile  (182 - 212 mg/dL) | 3rd tertile  (≥ 212 mg/dL) | *p*- value |
| --- | --- | --- | --- | --- | --- |
| All-cause mortality  Cases  Unadjusted HR (95% CI)  Model 1^a^  Adjusted HR (95% CI)  Model 2^b^  Adjusted HR (95% CI) | 4,402  1.33 (1.27 – 1.39)  1.29 (1.23 – 1.34)  1.13 (1.06 – 1.20) | < 0.001  < 0.001  < 0.001 | 3,291  1.00  1.00  1.00 | 3,422  1.05 (0.99 – 1.10)  1.03 (0.98 – 1.08)  1.15 (1.08 – 1.22) | 0.052  0.276  < 0.001 |
| CVD mortality  Cases  Unadjusted HR (95% CI)  Model 1^a^  Adjusted HR (95% CI)  Model 2^b^  Adjusted HR (95% CI) | 592  1.19 (1.06 – 1.34)  1.15 (1.02 – 1.30)  1.22 (1.05 – 1.41) | 0.004  0.020  0.010 | 494  1.00  1.00  1.00 | 658  1.34 (1.19 – 1.51)  1.29 (1.14 – 1.44)  1.17 (1.00 – 1.37) | < 0.001  < 0.001  0.054 |
| Cancer mortality  Cases  Unadjusted HR (95% CI)  Model 1^a^  Adjusted HR (95% CI)  Model 2^b^  Adjusted HR (95% CI) | 2,033  1.39 (1.30 – 1.48)  1.34 (1.26 – 1.44)  1.20 (1.09 – 1.31) | < 0.001  < 0.001  < 0.001 | 1,457  1.00  1.00  1.00 | 1,448  1.00 (0.93 – 1.08)  0.99 (0.92 – 1.07)  1.10 (1.00 – 1.21) | 0.947  0.793  0.062 |

TC, Total cholesterol; CVD, cardiovascular disease; HR, Hazard ratio; CI, Confidence interval

^a^ Adjusted for age and sex in model 1

^b^ Adjusted for age, sex, body mass index, baseline total cholesterol, systolic blood pressure, fasting blood glucose, hypertension, diabetes, Charlson comorbidity index, alcohol drinking, smoking status, disability and household income in model 2.
