# Supplementary material for: Association of change in total cholesterol level with mortality: A population-based study
Source: PLoS One. 2018 Apr 19;13(4):e0196030. doi: 10.1371/journal.pone.0196030 (PMC5908176; doi:10.1371/journal.pone.0196030)
Supplement: S2 Table — (DOCX) [file pone.0196030.s002.docx]

**S2 Table.** Stratified, multivariate-adjusted analysis of all-cause mortality risk by change in total cholesterol

| Baseline TC | 1st tertile  (< 182 mg/dL) | | | | | 2nd tertile  (182 - 212 mg/dL) | | | 3rd tertile  (≥ 212 mg/dL) | | |
| --- | --- | --- | --- | --- | --- | --- | --- | --- | --- | --- | --- |
| Follow-up TC ^a^ | 1st | | 2^nd^ | | 3^rd^ | 1st | 2^nd^ | 3^rd^ | 1st | 2^nd^ | 3^rd^ |
| Adjusted HR ^b^ |  | |  | |  |  |  |  |  |  |  |
| Age, years  40-64  (95% CI)  ≥ 65  (95% CI) | 1.35  (1.21 – 1.51)  1.20  (1.08 – 1.34) | 1.18  (1.04 – 1.34)  1.02  (0.90 – 1.15) | | 1.21  (1.01 – 1.44)  1.12  (0.95 – 1.31) | | 1.18  (1.05– 1.32)  1.14  (1.02– 1.28) | 1.00  1.00 | 1.01  (0.89 – 1.14)  1.06  (0.94 – 1.19) | 1.56  (1.32 – 1.82)  1.41  (1.21 - 1.64) | 1.15  (1.01 – 1.31)  1.14  (1.01 – 1.30) | 1.01  (0.89 – 1.15)  1.29  (1.14 – 1.45) |
| Sex  Men  (95% CI)  Women  (95% CI) | 1.28  (1.17 – 1.41)  1.29  (1.11 – 1.50) | 1.13  (1.02 – 1.25)  1.01  (0.85 – 1.20) | | 1.24  (1.09 - 1.43)  0.98  (0.78 – 1.23) | | 1.17  (1.07– 1.28)  1.14  (0.97 – 1.33) | 1.00  1.00 | 1.06  (0.96 – 1.17)  0.96  (0.82– 1.12) | 1.55  (1.37 – 1.76)  1.27  (1.01 – 1.58) | 1.22  (1.09 – 1.35)  0.99  (0.83 – 1.17) | 1.13  (1.02 - 1.25)  1.13  (0.97– 1.32) |
| BMI, kg/m^2^  < 25  (95% CI)  ≥ 25  (95% CI) | 1.25  (1.14 – 1.36)  1.33  (1.14 – 1.56) | 1.07  (0.96 – 1.18)  1.19  (0.99 – 1.42) | | 1.18  (1.03 – 1.35)  1.11  (0.88 – 1.41) | | 1.12  (1.02 – 1.23)  1.30  (1.11 –1.52) | 1.00  1.00 | 0.97  (0.88 – 1.08)  1.18  (1.09 – 1.38) | 1.50  (1.32 – 1.71)  1.36  (1.09 – 1.71) | 1.16  (1.04 – 1.29)  1.11  (0.94 – 1.32) | 1.15  (1.04 – 1.27)  1.12  (0.95 – 1.32) |
| Smoking status  Never  (95% CI)  Former  (95% CI)  Current  (95% CI) | 1.26  (1.14 – 1.40)  1.28  (1.00 – 1.63)  1.30  (1.14 – 1.49) | 1.10  (0.98 – 1.24)  0.91  (0.69 – 1.21)  1.16  (0.99 – 1.35) | | 1.03  (0.87 – 1.21)  1.12  (0.77 – 1.61)  1.43  (1.17 – 1.75) | | 1.20  (1.08– 1.33)  1.07  (0.83 – 1.38)  1.10  (0.96 – 1.26) | 1.00  1.00  1.00 | 1.05  (0.94 – 1.18)  1.03  (0.79 – 1.35)  1.00  (0.86 – 1.17) | 1.58  (1.36 – 1.82)  1.67  (1.19 – 2.34)  1.24  (1.01 – 1.51) | 1.16  (1.03 – 1.31)  1.22  (0.92 – 1.62)  1.09  (0.93 – 1.27) | 1.23  (1.10 - 1.38)  1.22  (0.93 – 1.60)  0.98  (0.84 – 1.14) |
| Drinking  No  (95% CI)  Yes  (95% CI) | 1.18  (1.06 – 1.31)  1.40  (1.25 – 1.57) | 1.04  (0.92 – 1.17)  1.18  (1.04 – 1.35) | | 1.01  (0.86 - 1.19)  1.39  (1.17 – 1.66) | | 1.12  (1.01– 1.25)  1.20  (1.06 –1.35) | 1.00  1.00 | 1.06  (0.94 – 1.18)  1.00  (0.88– 1.15) | 1.36  (1.17 – 1.59)  1.59  (1.35 – 1.88) | 1.09  (0.97 – 1.24)  1.21  (1.06 – 1.39) | 1.20  (1.07 - 1.35)  1.06  (0.93– 1.21) |
| Household income  1^st^ quinrtile  (95% CI)  2^nd^ quintile  (95% CI)  3^rd^ quintile  (95% CI)  4^th^ quintile  (95% CI)  5^th^ quintile  (95% CI) | 1.05  (0.88 – 1.24)  1.18  (0.99 – 1.41)  1.50  (1.23 – 1.82)  1.51  (1.25 – 1.81)  1.28  (1.10 – 1.50) | 0.91  (0.75 – 1.10)  1.12  (0.92 – 1.37)  1.15  (0.92 – 1.44)  1.20  (0.97 – 1.48)  1.14  (0.96 – 1.36) | | 1.23  (0.97 - 1.54)  1.16  (0.89 – 1.51)  1.06  (0.77 – 1.46)  1.16  (0.87 – 1.54)  1.11  (0.86 – 1.43) | | 1.01  (0.84 – 1.20)  1.10  (0.92 – 1.32)  1.13  (0.92 – 1.39)  1.33  (1.10 – 1.60)  1.23  (1.05 – 1.44) | 1.00  1.00  1.00  1.00  1.00 | 1.08  (0.90 – 1.29)  1.04  (0.85– 1.26)  0.78  (0.62 – 0.99)  1.10  (0.90 – 1.35)  1.08  (0.92 – 1.28) | 1.66  (1.32 – 2.07)  1.40  (1.09 – 1.80)  1.19  (0.88 – 1.60)  1.52  (1.17 – 1.97)  1.49  (1.19 – 1.87) | 1.18  (0.97 – 1.44)  1.02  (0.83 – 1.26)  1.03  (0.81 – 1.31)  1.13  (0.91 – 1.39)  1.30  (1.09 – 1.55) | 1.15  (0.96 - 1.39)  0.93  (0.76 – 1.14)  1.03  (0.83 – 1.29)  1.18  (0.96 – 1.44)  1.36  (1.15 – 1.60) |
| CCI  < 4  (95% CI)  ≥ 4  (95% CI) | 1.26  (1.16 – 1.38)  1.36  (1.11 – 1.66) | 1.12  (1.02 – 1.23)  1.03  (0.82 – 1.29) | | 1.12  (0.98 - 1.28)  1.37  (1.05 – 1.79) | | 1.14  (1.05– 1.25)  1.24  (1.01 –1.53) | 1.00  1.00 | 0.99  (0.91 – 1.01)  1.25  (1.00 – 1.56) | 1.40  (1.24 – 1.59)  1.81  (1.38 – 2.39) | 1.11  (1.01 – 1.22)  1.31  (1.02 – 1.67) | 1.07  (0.98 - 1.18)  1.57  (1.26 – 1.96) |
| Hypertension  No  (95% CI)  Yes  (95% CI) | 1.32  (1.22 – 1.44)  1.03  (0.84 – 1.27) | 1.11  (1.01 – 1.22)  1.05  (0.84 – 1.31) | | 1.19  (1.04 - 1.35)  1.05  (0.78 – 1.42) | | 1.19  (1.09 – 1.29)  1.01  (0.82 – 1.25) | 1.00  1.00 | 1.02  (0.93 – 1.12)  1.07  (0.87 – 1.32) | 1.48  (1.31 – 1.67)  1.44  (1.09 – 1.91) | 1.16  (1.05 – 1.28)  1.08  (0.85 – 1.36) | 1.12  (1.02 - 1.23)  1.30  (1.05 – 1.61) |
| Diabetes  No  (95% CI)  Yes  (95% CI) | 1.30  (1.20 – 1.41)  1.01  (0.77 – 1.33) | 1.10  (1.00 – 1.20)  1.10  (0.81 – 1.50) | | 1.14  (1.00 – 1.29)  1.34  (0.92 – 1.95) | | 1.17  (1.07 – 1.27)  1.07  (0.81 – 1.41) | 1.00  1.00 | 1.03  (0.94 – 1.13)  1.00  (0.74 – 1.36) | 1.49  (1.49 – 1.67)  1.32  (0.92 – 1.89) | 1.16  (1.05 – 1.27)  1.06  (0.77 – 1.46) | 1.15  (1.05 – 1.26)  1.12  (0.83 – 1.52) |

TC, Total cholesterol; CCI, Charlson comorbidity index; HR, Hazard ratio; CI, Confidence interval

^a^ Cholesterol levels at follow-up were divided into three groups according to tertiles; 1^st^ tertile (< 181 mg/dL), 2^nd^ tertile (181 – 210 mg/dL) and 3^rd^ tertile (≥ 211 mg/dL).

^b^ Adjusted for age, sex, body mass index, baseline total cholesterol, systolic blood pressure, fasting blood glucose, hypertension, diabetes, Charlson comorbidity index, alcohol drinking, smoking status, disability and household income.
