# Supplementary material for: Association of change in total cholesterol level with mortality: A population-based study
Source: PLoS One. 2018 Apr 19;13(4):e0196030. doi: 10.1371/journal.pone.0196030 (PMC5908176; doi:10.1371/journal.pone.0196030)
Supplement: S3 Table — (DOCX) [file pone.0196030.s003.docx]

**S3 Table**. **The association between mortality and cholesterol change among subjects with statin medication** ^a^

| Baseline TC | 1st tertile  (< 182 mg/dL) | | | | | 2nd tertile  (182 - 212 mg/dL) | | | | 3rd tertile  (≥ 212 mg/dL) | | | |
| --- | --- | --- | --- | --- | --- | --- | --- | --- | --- | --- | --- | --- | --- |
| Follow-up TC | 1st | 2nd | | | 3rd | 1st | | 2nd | 3rd | 1st | 2nd | | 3rd |
| All-cause mortality  aHR ^b^  (95% CI)  *p*-value | 1.59  (1.14 – 2.21)  0.006 | | 1.30  (0.90 – 1.88)  0.168 | 0.99  (0.62 – 1.61)  0.987 | | 1.38  (0.99 – 1.94)  0.061 | 1.00 | | 1.39  (0.98 – 1.97)  0.062 | 1.34  (0.89 – 2.02)  0.155 | 0.86  (0.57 – 1.29)  0.464 | 1.14  (0.79 – 1.64)  0.475 | |
| CVD mortality  aHR ^b^  (95% CI)  *p*-value | 2.62  (1.20 – 5.76)  0.016 | | 2.72  (1.19 – 6.24)  0.018 | 1.86  (0.65 – 5.27)  0.245 | | 1.13  (0.45 – 2.81)  0.792 | | 1.00 | 2.58  (1.18 – 5.64)  0.017 | 1.08  (0.39 – 2.99)  0.888 | 0.59  (0.20 – 1.73)  0.337 | | 1.04  (0.43 – 2.50)  0.939 |
| Cancer mortality  aHR ^b^  (95% CI)  *p*-value | 1.40  (0.84 – 2.31)  0.196 | | 1.11  (0.63 – 1.98)  0.716 | 0.81  (0.37 – 1.75)  0.586 | | 1.46  (0.89 – 2.41)  0.135 | | 1.00 | 1.09  (0.63 – 1.88)  0.767 | 1.24  (0.67 – 2.29)  0.500 | 0.70  (0.37 – 1.33)  0.275 | | 1.18  (0.69 – 2.02)  0.546 |

TC, Total cholesterol; CVD, cardiovascular disease; HR, Hazard ratio; CI, Confidence interval

^a^ 15,140 Subjects who have claim record more than 30 cumulative defined daily dose (DDD) were defined as statin users through 2002-2006.

^b^ Adjusted for age, sex, body mass index, baseline total cholesterol, systolic blood pressure, fasting blood glucose, hypertension, diabetes, Charlson comorbidity index, alcohol drinking, smoking status, disability and household income.
