# Supplementary figures and images for: Association of change in total cholesterol level with mortality: A population-based study
Source: PLoS One. 2018 Apr 19;13(4):e0196030. doi: 10.1371/journal.pone.0196030 (PMC5908176; doi:10.1371/journal.pone.0196030)

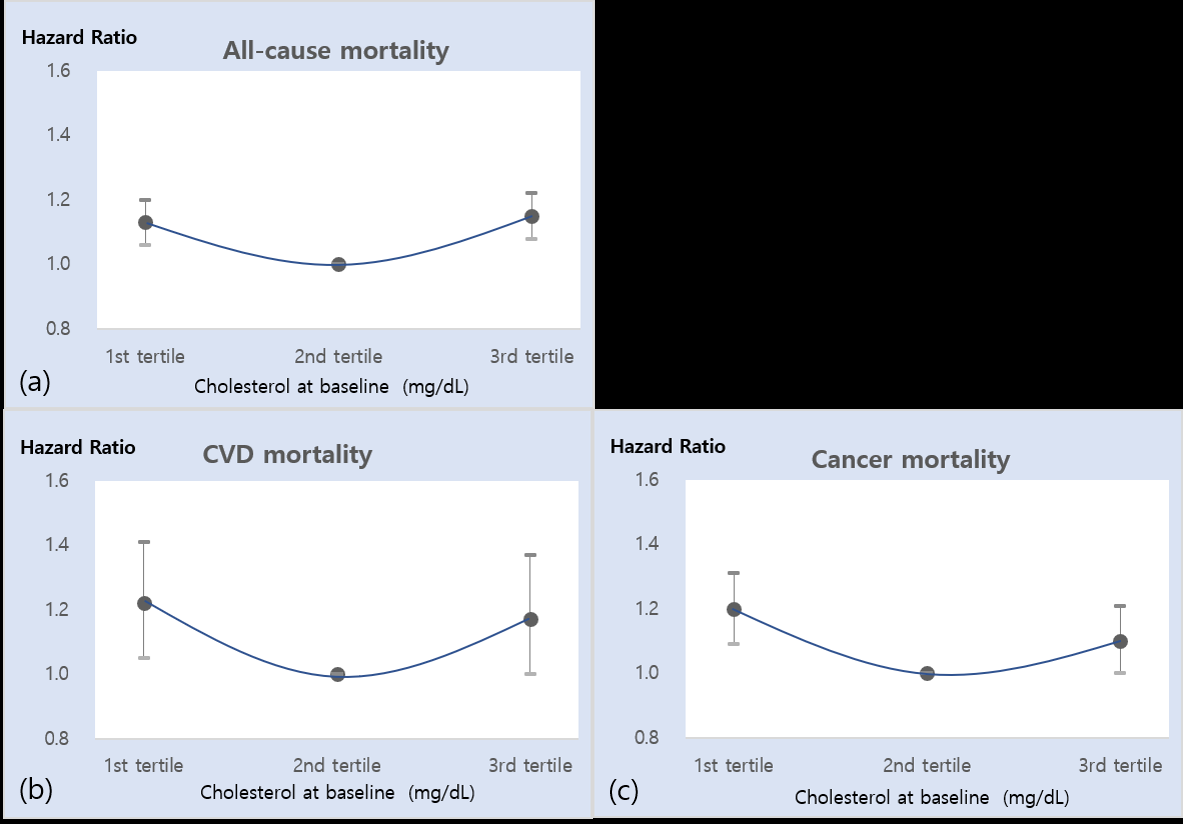

Supplement: S1 Fig — (a) All-cause mortality (b) Cardiovascular disease mortality and (C) Cancer mortality according to the tertiles of baseline cholesterol showed U-shaped association. (TIF) [file pone.0196030.s004.tif]
